# Association between retinal thickness and disease characteristics in adult epilepsy: A cross‐sectional OCT evaluation

**DOI:** 10.1002/epi4.12859

**Published:** 2023-11-22

**Authors:** Luisa Delazer, Han Bao, Michael Lauseker, Livia Stauner, Georg Nübling, Julian Conrad, Soheyl Noachtar, Joachim Havla, Elisabeth Kaufmann

**Affiliations:** ^1^ Epilepsy Center, Department of Neurology LMU University Hospital, LMU Munich Munich Germany; ^2^ Institute for Medical Information Processing, Biometry, and Epidemiology Ludwig Maximilians University Munich Germany; ^3^ Institute for Statistics Munich Germany; ^4^ Department of Neurology LMU University Hospital, LMU Munich Munich Germany; ^5^ German Center for Neurodegenerative Diseases Munich Germany; ^6^ Division for Neurodegenerative Diseases Universitätsmedizin Mannheim, University of Heidelberg Heidelberg Germany; ^7^ Institute of Clinical Neuroimmunology LMU Hospital LMU Hospital, Ludwig Maximilians University Munich Germany

**Keywords:** anti‐seizure medication, brain atrophy, disease burden, focal to bilateral tonic clonic seizures, optical coherence tomography, sexual dimorphism

## Abstract

**Objective:**

Thinning of the peripapillary retinal nerve fiber layer (p‐RNFL), as measured by optical coherence tomography (OCT), was recently introduced as a promising marker for cerebral neuronal loss in people with epilepsy (PwE). However, its clinical implication remains to be elucidated. We thus aimed to (1) systematically characterize the extent of the retinal neuroaxonal loss in a broad spectrum of unselected PwE and (2) to evaluate the main clinical determinants.

**Methods:**

In this prospective study, a spectral‐domain OCT evaluation was performed on 98 well‐characterized PwE and 85 healthy controls (HCs) (18–55 years of age). All inner retinal layers and the total macula volume were assessed. Group comparisons and linear regression analyses with stepwise backward selection were performed to identify relevant clinical and demographic modulators of the retinal neuroaxonal integrity.

**Results:**

PwE (age: 33.7 ± 10.6 years; 58.2% female) revealed a significant neuroaxonal loss across all assessed retinal layers (global pRNFL, *P* = 0.001, Δ = 4.24 μm; macular RNFL, *P* < 0.001, Δ = 0.05 mm^3^; ganglion cell inner plexiform layer, *P* < 0.001, Δ = 0.11 mm^3^; inner nuclear layer, INL, *P* = 0.03, Δ = 0.02 mm^3^) as well as significantly reduced total macula volumes (TMV, *P* < 0.001, Δ = 0.18 mm^3^) compared to HCs (age: 31.2 ± 9.0 years; 57.6% female). The extent of retinal neuroaxonal loss was associated with the occurrence and frequency of tonic–clonic seizures and the number of antiseizure medications, and was most pronounced in male patients.

**Significance:**

PwE presented an extensive retinal neuroaxonal loss, affecting not only the peripapillary but also macular structures. The noninvasive and economic measurement via OCT bears the potential to establish as a practical tool to inform patient management, as the extent of the retinal neuroaxonal loss reflects aspects of disease severity and sex‐specific vulnerability.

**Plain Language Summary:**

The retina is an extension of the brain and closely connected to it. Thus, cerebral alterations like atrophy reflect also on the retinal level. This is advantageous, as the retina is easily accessible and measureable with help of the optical coherence tomography. Here we report that adults with epilepsy have a significantly thinner retina than healthy persons. Especially people with many big seizures and a lot of medications have a thinner retina. We propose that measurement of the retina can be useful as a marker of disease severity and to inform patient management.

**Key points**

- Epilepsy was associated with extensive retinal neuroaxonal loss affecting peripapillary (G‐pRNFL) and macular structures (mRNFL, GCIP, INL, and TMV).
- The extent of the retinal thinning reflected aspects of disease activity and severity, including the frequency of tonic–clonic seizures and the number of antiseizure medications.
- The study findings suggest a sex‐specific vulnerability to neuroaxonal loss to the detriment of male patients.

## 1 INTRODUCTION

People with epilepsy (PwE) do not only suffer from seizures but also from adverse long‐term consequences like brain atrophy.^1^ However, a sensitive and clinically feasible marker to estimate and monitor the individual disease sequelae is still missing. Thus, patient management is still mainly based on seizure diaries which are known to be highly incorrect.^2^

Recently, the thickness/volume of the inner retinal layers was suggested as a reliable surrogate marker for cerebral axonal injury in healthy volunteers as well as several neurological conditions: the community based (n = 2872) Rhineland study, for example, reported an association between the volumes of the inner retina and the total brain volume, the cerebral gray and white matter volume, as well as the hippocampal volume.^3^ In inflammatory or neurodegenerative disorders like multiple sclerosis, Alzheimer's and Parkinson's disease, the retinal neuroaxonal alterations further indicated disease activity, severity, and progression.^4^, ^5^, ^6^, ^7^ Similar patterns of retinal axonal and neuronal loss were also described in professional collision sports athletes, accompanied by impaired visual function and reduced quality of life.^8^

Of note, the retina and the brain are developmentally evolved from the same neuronal tissue. Thus, they have morphological and physiological similarities and direct synaptic connections exist. Cerebral disorders can thus reflect on retinal level due to trans‐synaptic axonal degeneration.^9^ In this context, the innermost retinal layer, the retinal nerve fiber layer (RNFL), has been most extensively studied so far. The RNFL contains the unmyelinated axons of retinal ganglion cells which project to the lateral geniculate nucleus and thus best reflect the integrity of the cerebral white matter. However, it is already known that the trans‐synaptic retrograde atrophic processes involve not only the RNFL but also the adjacent layers of the inner retina, i.e., the ganglion cell layer (GCL) and inner plexiform layer (IPL), and typically stop at the inner nuclear layer (INL).^9^, ^10^ Precise measurements of the thickness/volume of the inner retinal layers are performed using optical coherence tomography (OCT), which is a noninvasive, easy‐to‐use, and economic technique that can be repeated as often as required.

In PwE, first cross‐sectional OCT evaluations were also promising. They described signs of retinal neuroaxonal loss in adults with epilepsy,^11^, ^12^, ^13^ which correlated with disease duration as well as intellectual disability.^12^ Retinal axonal loss was advanced in PwE who proved to be drug‐resistant, received polypharmacotherapy, or were treated with certain antiseizure medications (ASM), namely vigabatrin, ethosuximide, primidone, phenytoin, topiramate, and valproate.^12^, ^14^, ^15^, ^16^, ^17^ Besides, the main clinical and demographic drivers of retinal thinning in epilepsy are still unknown. Of note, previous OCT studies in epilepsy focused on the peripapillary RNFL only,^12^, ^15^, ^16^, ^18^ were all but one small‐scaled,^11^, ^14^ were mostly restricted to certain syndromes^18^ or ASM subgroups,^15^, ^16^, ^17^ and did not take seizure semiology and frequency into account. Furthermore, none of the studies controlled for the time since the last change in ASM and last experienced seizure, and thus disregarded putative acute effects^8^, ^19^, ^20^: There is ample data that ASM changes, especially sodium channel blockers, can cause focal cerebral edema upon quick withdrawal or reintroduction^21^ and co‐occurring retinal changes seem possible. Further, tonic–clonic seizures (TCS) may lead to traumatic optic neuropathy and are followed by a blood–brain barrier leakage.^22^, ^23^ This might have biased the OCT measurements and thus could have contributed to the inconsistent study results in the literature.

This cross‐sectional cohort study aimed to (1) confirm the previously reported RNFL loss in an unselected cohort of consecutive adults with epilepsy. The cohort was comprised of PwE with a broad range of disease severity, including PwE with only one prior seizure up to people with drug‐resistant epilepsy, as well as PwE who underwent resective surgery and/or neurostimulation. Hypothetically, PwE present a significantly thinner RNFL compared with healthy controls (HC). Further, we aimed to explore (2) the extent of the retinal changes by investigating OCT measures of all inner retinal layers and the total macula volume, as well as (3) the impact of disease characteristics on retinal changes.

## 2 METHODS

### 2.1 Participants and recruitment

Consecutive adults diagnosed with epilepsy were prospectively recruited from the epilepsy outpatient clinic and the epilepsy monitoring unit at the University Hospital, LMU Munich, Germany, from February 2021 to August 2021. HC were recruited from hospital staff and acquaintances.

Only PwE and HC aged between 18 and 55 years with the physical ability to take part in the study examinations were enrolled. Exclusion criteria for both groups encompassed a refractive error of more than +/− 4.5 diopters mean sphere or more than 2.5 diopters cylinder, lesions in the central visual pathway, history of eye surgery, or known ocular disease as listed in the OSCAR‐IB consensus criteria such as macular degeneration, glaucoma, or history of optic neuritis, as well as current or former vigabatrin intake.^24^ PwE and HC were also excluded due to pregnancy, diabetes mellitus, untreated arterial hypertension, current or former drug abuse, cognitive disability (as mentioned in medical records and/or IQ < 70 in neuropsychological testing), and neurological disease other than epilepsy and migraine. PwE with a change in antiseizure medication within the last 14 days or a recent TCS within the last 48 hours were not included in the study to avoid a potential bias due to assumed acute effects.^8^, ^19^, ^20^ A total of 108 PwE and 90 HC underwent the study protocol. Ten of them were excluded from the analysis due to a delayed identification of exclusion reasons (n = 9): history of vigabatrine intake, history of drug abuse, history of subarachnoidal bleeding (n = 2), history of severe concussion and subdural hemorrhage, increased optic nerve sheath diameter, LGI1 autoimmune encephalitis, postsurgical visual field defects (n = 2), or poor OCT scan quality (n = 1). Healthy controls whose OCT scan quality was low (n = 2), or whose OCT measures differed more than 2.5 standard deviations from the group mean (outliers, n = 3) were excluded as a retinal pathological change cannot be excluded. Four PwE had no reliable seizure records and were thus not considered for the multivariable models.

### 2.2 Clinical and demographic parameters

Upon study participation, demographic, and clinical data with possible impact on retinal layer measurements and visual function were obtained from PwE and HC via interview, including sex, age, ethnicity, body mass index (BMI), and presence of treated arterial hypertension. For PwE, a detailed history of medications, including the number of current and total ASM (the latter including current and prior ASM), as well as other medication and for HC the intake of oral contraception was obtained. Furthermore, years of education, disease duration, disease etiology and seizure semiology, history of psychogenic non‐epileptic seizures, history of brain surgeries, and use of implantable stimulation devices were recorded. Epileptic seizure were classified according to the 2017 guideline of the International League Against Epilepsy (ILAE).^25^ The frequency of different seizure types was determined based on medical records, seizure calendars, as well as interviews with PwE and/or companions. The number of all seizures in the last year excluding isolated auras, as well as the highest annual frequency of TCS were assessed as indicators for the individual disease activity. The highest annual frequency of TCS was obtained from medical records or patients' reports.

### 2.3 Optical coherence tomography

A Heidelberg Engineering spectral‐domain optical coherence tomography (SD‐OCT, SPECTRALIS; Heidelberg Engineering) with automatic real‐time (ART) averaging was used for measuring thickness and volumes of retinal layers. The thickness of the global peripapillary RNFL (G‐pRNFL) was measured in a peripapillary scan with automatic eye tracking (12°, 3.5 mm ring, 50 ≤ ART ≤ 100). The volume of the macular RNFL (mRNFL), the ganglion cell inner plexiform layer (GCIP), INL, and the total macula volume (TMV) were analyzed in a cylinder scan of six millimeters diameter around the fovea (20° × 20°, 25 vertical B‐scans, 20 ≤ ART ≤ 49). The semi‐automatic segmentation of all layers was performed using Eye Explorer (version 1.9.10.0) with viewing module 6.3.4.0 (Heidelberg Engineering). For further statistical analyses, an average measure of the left and right eye was used, and only peripapillary scans with a quality >20 (as defined by SPECTRALIS) and an ART ≥90 and macula scans with a quality >20 and ART >40 were included. All OCT examinations were performed in the NeuroVisionLab of the Institute of Clinical Neuroimmunology, LMU Hospital. All participants were examined by the same investigator (LD). LD also performed quality control according to OSCAR‐IB criteria and segmentation of all retinal layers.^24^

### 2.4 Statistical analysis

Statistical analyses were performed using SPSS version 26 (IBM SPSS Statistics) and R 4.2.0 (©The R Foundation). Group comparisons were performed via Chi‐square test for categorial variables and via two sample independent two‐tailed *t*‐test or ANOVA for continuous variables followed by Scheffé corrected post hoc tests in case of significant results. Group comparisons of retinal measures (independent *t*‐tests and global ANOVAs) were Benjamini–Hochberg corrected.

An explorative multiple linear model was estimated for all OCT measures. Backward stepwise selection was performed using the Akaike information criterion (AIC) to decide which model was best. The following variables were selected based on the literature, as they were described to be associated with the retinal^12^, ^14^, ^26^, ^27^ and/or cerebral volume or thickness^28^, ^29^, ^30^, ^31^, ^32^, ^33^ and entered as possible predictors: age, sex, disease duration, highest annual frequency of TCS, number of current ASM, and neurostimulation therapy. A multilinear regression model was also estimated for HCs, which included age and sex as possible predictors. Descriptive data is presented as mean and standard deviation or median and interquartile range (IQR). A *P*‐value <0.05 was considered statistically significant.

### 2.5 Standard protocol approvals, registrations, and patient consents

This study was approved by the local ethics committee (LMU Munich, No. 21‐0014), and all participants provided written informed consent before study participation. The authors followed the STROBE guideline for the preparation of the manuscript.

## 3 RESULTS

### 3.1 Demographic and clinical characteristics

The clinical and demographic characteristics of the epilepsy cohort (n = 98) and the HC group (n = 85) are summarized in Table 1. The epilepsy cohort had a mean disease duration of 12.0 ± 11.8 years and encompassed 19 (19.4%) people with genetic generalized epilepsy, 28 (28.6%) with temporal lobe epilepsy (TLE), 15 (15.3%) with frontal lobe epilepsy (FLE), and 36 (36.7%) people with multifocal or a yet unclassified epilepsy. Most (n = 79, 80.6%) PwE had experienced at least one TCS in their life, while 19.4% (n = 19) exclusively reported seizures without bilateral tonic–clonic convulsions. Seven (7.1%) PwE had experienced only one seizure so far (all of which had a bilateral tonic–clonic semiology and an additional EEG and/or MRI pathology).

**TABLE 1:** Cohort characteristics.

	People with epilepsy (n = 98)	Healthy controls (n = 85)	*P*‐value
Sex			0.94^c^
Female	57 (58.2%)	49 (57.6%)	
Male	41 (41.8%)	36 (42.4%)	
Ethnicity			0.56^c^
Caucasian	97 (99.0%)	83 (97.6%)	
Afro‐American	1 (1.0%)	1 (1.2%)	
Chinese	0 (0.0%)	1 (1.2%)	
Age [years]	33.7 ± 10.6	31.2 ± 9.0	0.09^d^
Years of education	11.9 ± 2.9	15.6 ± 2.9	<0.001^d^
Smoker			0.36^c^
Yes	22 (22.4%)	16 (18.8%)	
No	76 (77.6%)	69 (81.2%)	
Body mass index [kg/m^2^]	25.4 ± 4.9	23.7 ± 4.1	<0.001^d^
Disease duration [years]	12.0 ± 11.8		
Epilepsy classification			
Genetic generalized epilepsy	19 (19.4%)		
Temporal lobe epilepsy	28 (28.6%)		
Frontal lobe epilepsy	15 (15.3%)		
Other (multifocal, undefined)	36 (36.7%)		
Focal onset epilepsies			
Left hemispheric onset	31 (31.6%)		
Right hemispheric onset	20 (20.4%)		
Other (bihemispheric, undefined)	28 (28.6%)		
Seizure semiology (number of people with epilepsy)			
Focal aware seizure (FAS)	22 (22.4%)		
Focal impaired awareness seizure (FIAS)	26 (26.5%)		
Focal to bilateral tonic–clonic seizure (FBTCS)	66 (67.3%)		
Generalized tonic–clonic seizure (GTCS)	13 (13.3%)		
Generalized myoclonic seizure	5 (5.1%)		
Generalized non‐motor seizure	8 (8.2%)		
Other (unknown onset / unclassified)	15 (1.0%)		
Overall seizure frequency in the last year (n = 94^b^)			
Median [IQR]	9.5 [1–48]		
Mean ± std	46.8 ± 94.4		
Min–max	0–500^a^		
Frequency of all seizures except TCS in the last year (n = 94^b^)			
Median [IQR]	1.5 [0–36]		
Mean ± std	41.4 ± 94.8		
Min–max	0–500^a^		
Maximum annual frequency of TCS (n = 98)			
Median [IQR]	2 [1–6]		
Mean ± std	7.2 ± 14.9		
Min–max	0–90		
Latency of acute (<3 months) TCS (n = 19) and study examination [weeks]			
Median [IQR]	2.0 [0.4–6.0]		
Mean ± std	3.3 ± 3.0		
Latency of remote (3–12 months) TCS (n = 37) and study examination [months]			
Median [IQR]	6.0 [4.5–10.0]		
Mean ± std	7.2 ± 3.2		
Number of current ASM			
Mean ± std	1.9 ± 1.1		
0 ASM	4		
1 ASM	39		
2 ASM	33		
3 ASM	14		
4 ASM	6		
5 ASM	2		
Number of total ASM (i.e., current + prior)			
mean ± std	4.07 ± 3.03		
Surgical history			
Prior resective surgery/neurostimulation current	22/1		
VNS/DBS treatment	7/3		

Abbreviations: ASM, anti‐seizure medication; DBS, deep brain stimulation; IQR, inter‐quartile range; max, maximum; min, minimum; std, standard deviation; TCS, tonic–clonic seizure; VNS, vagal nerve stimulation.

^a^ One subject had FIAS several times a day.

^b^ Four patients were excluded due to imprecise seizure documentation.

^c^ Chi‐square test.

^d^ Independent sample *t*‐test (two‐tailed).

### 3.2 Retinal measures in epilepsy versus healthy controls

**FIGURE 1:**
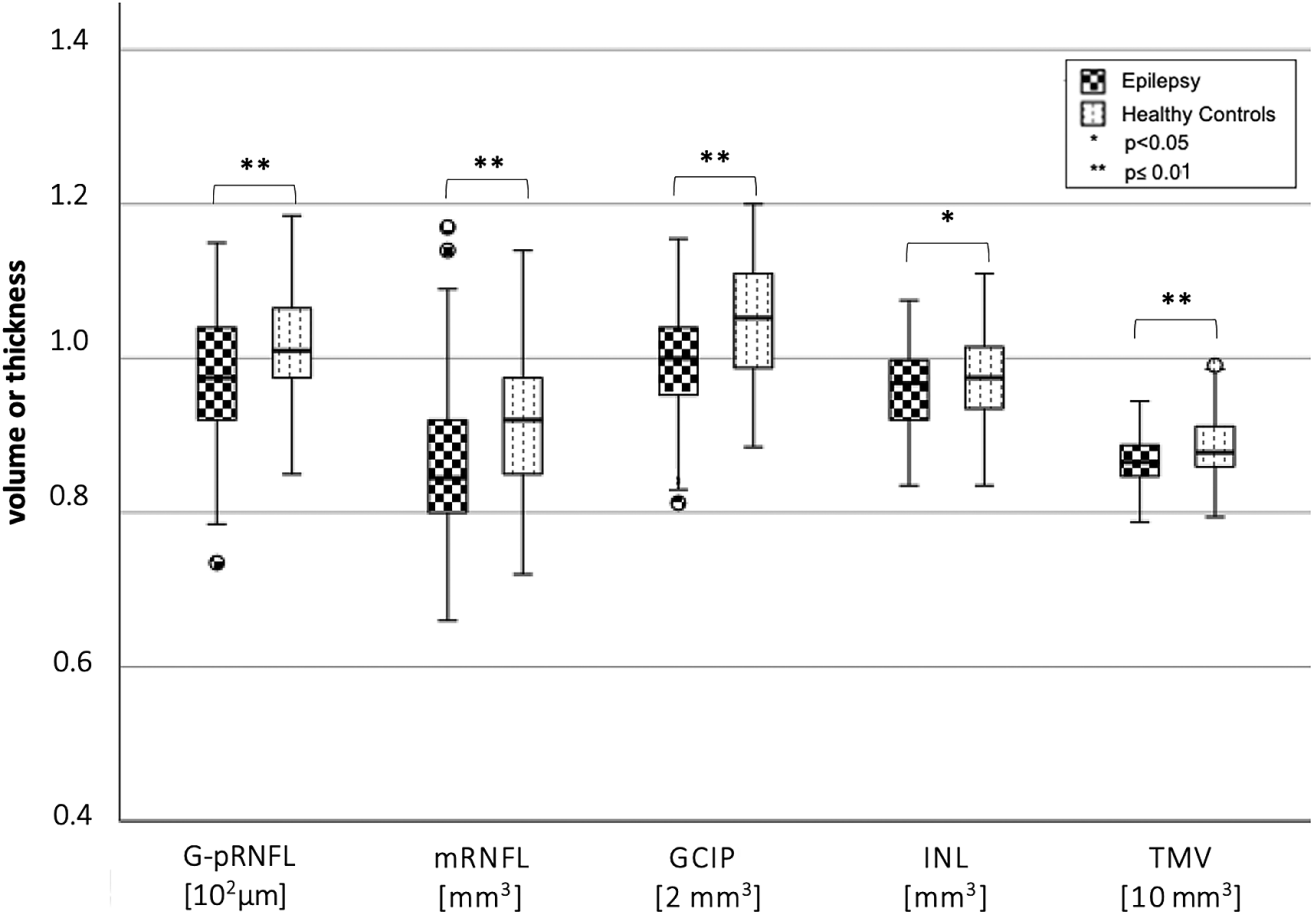
Thickness and volumes of retinal layers in people with epilepsy (PwE) and healthy controls (HC). The box‐plot figure represents the median (plus first and third quartile, minimum and maximum) thickness and volume of the different retinal layers in PwE (n = 98) compared to HC (n = 85). Outliers are indicated as separate dots. PwE had a significantly thinner global retinal nerve fiber layer (G‐pRNFL) as well as lower volume measures for the macular RNFL (mRNFL), ganglion cell inner plexiform layer (GCIP), inner nuclear layer (INL), and lower total macular volumes (TMV). The respective means and standard deviations are summarized in Table 2.

**FIGURE 2:**
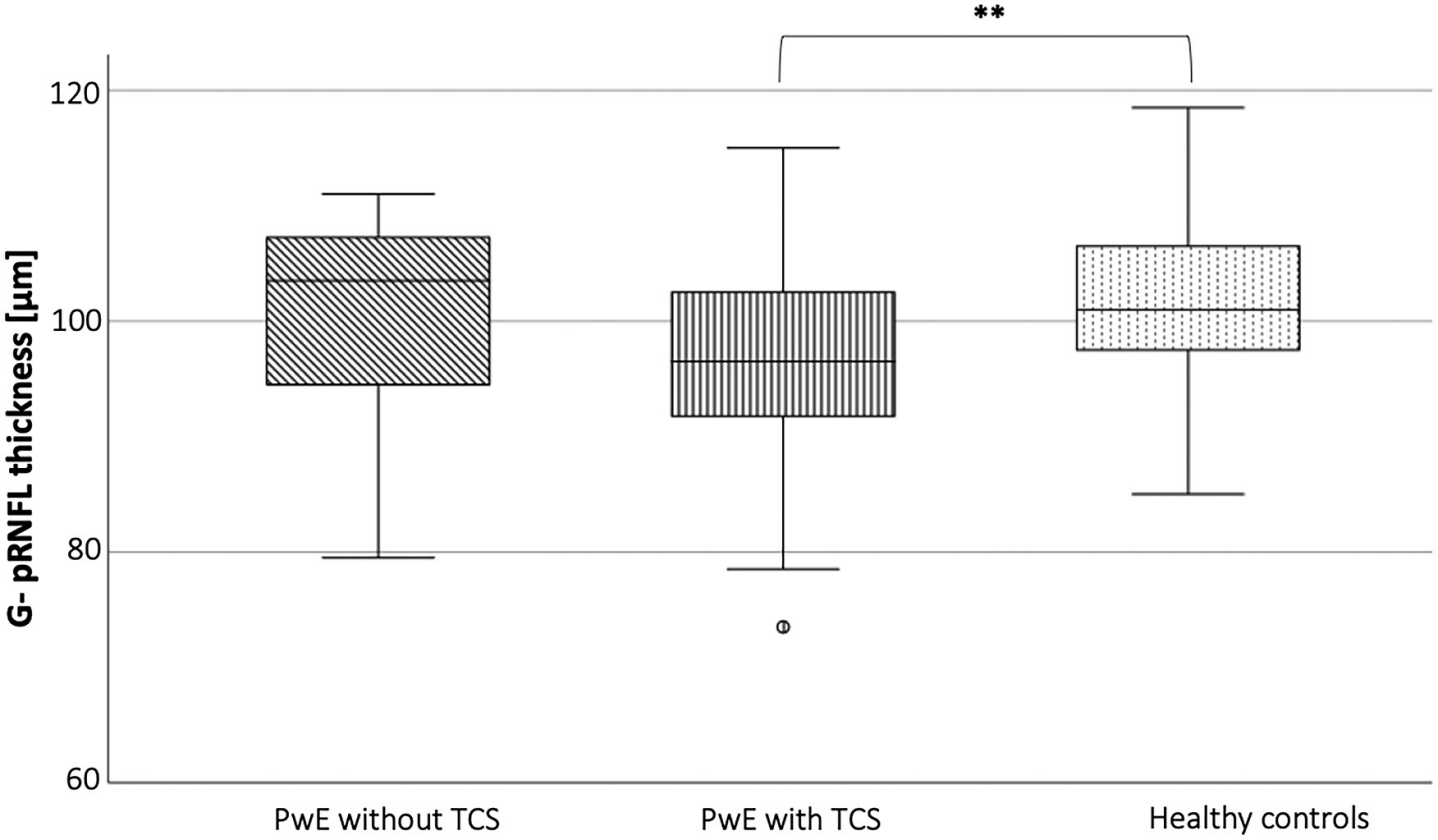
Thickness of global peripapillary retinal nerve fiber layer (G‐pRNFL) with regard to the occurrence of tonic–clonic seizures. The box‐plot figure visualizes the median, the first and third quartile, minimum and maximum thickness of the G‐pRNFL in people with epilepsy (PwE) with (n = 79) and without (n = 19) tonic–clonic seizures (TCS) in comparison to healthy controls (HC, n = 85). **P < 0.01.

PwE (n = 98) had significantly decreased volumes of all assessed inner retinal layers as well as a significantly reduced total macula volume compared to HC (n = 85) (Table 2A, Figure 1). The two groups differed in BMI and years of education (Table 1), but neither BMI nor age‐corrected years of education were significantly associated with the retinal measures in any of the two groups. Of note, a significant retinal neuroaxonal loss was observed in the subgroup of PwE with a history of at least one TCS (n = 79, age: 32.5 ± 10.5 years, 45 female; TCS per year: mean ± std 8.9 ± 16.2; median 2 [1–10]) compared to HC (Table 2B, Figure 2). In contrast, PwE who never had a TCS (n = 19, age: 38.7 ± 10.2 years, 12 female) did not significantly differ in their retinal measures from a HC subgroup with comparable age and sex distribution (n = 19, age: 38.1 ± 9.7 years, 12 female) nor from the subgroup with at least one TCS. The subgroup of PwE without TCS had a comparable disease duration and number of ASM like those with TCS and were even significantly older.

**TABLE 2:** Group comparison regarding the mean measures of the retinal layers and the total macula volume in (A) people with epilepsy (PwE) and healthy controls (HC) and (B) PwE with no history of a tonic–clonic seizure (PwE without TCS), PwE with a history of at least one tonic–clonic seizure (PwE with TCS) and HC.

A				
	PwE (n = 98)	HC (n = 85)	Independent *t*‐test (two‐tailed)	
			*P*‐value	Δ
G‐pRNFL [μm]	97.30 ± 8.97	101.54 ± 6.91	0.001	4.24
mRNFL [mm^3^]	0.86 ± 0.09	0.91 ± 0.09	0.002	0.05
GCIP [mm^3^]	1.99 ± 0.14	2.10 ± 0.15	0.003	0.11
INL [mm^3^]	0.96 ± 0.05	0.98 ± 0.06	0.03	0.02
TMV [mm^3^]	8.66 ± 0.32	8.84 ± 0.37	0.005	0.18

B									
	PwE without TCS (n = 19)		PwE with TCS (n = 79)		HC (n = 85)	ANOVA global test statistics		Post‐hoc tests	
	Δ to HC		Δ to HC		*F* (2, 180)		*P*‐value	Significant group differences	*P*‐value
G‐pRNFL [μm]	99.87 ± 10.14	1.67	96.68 ± 8.62	4.86	101.54 ± 6.91	7.52	0.002	PWE with TCS < HC	<0.001
mRNFL [mm^3^]	0.86 ± 0.12	0.05	0.86 ± 0.09	0.05	0.91 ± 0.09	8.23	0.003	PWE with TCS < HC	<0.001
GCIP [mm^3^]	2.02 ± 0.17	0.08	1.99 ± 0.13	0.11	2.10 ± 0.15	12.53	0.005	PWE with TCS < HC	<0.001
INL [mm^3^]	0.95 ± 0.06	0.03	0.96 ± 0.05	0.02	0.98 ± 0.06	4.48	0.03	n.s.	
TMV [mm^3^]	8.69 ± 0.38	0.15	8.65 ± 0.30	0.19	8.84 ± 0.37	6.42	0.003	PWE with TCS < HC	0.003

Abbreviations: FLE, frontal lobe epilepsy; GCIP, ganglion cell inner plexiform layer; G‐pRNFL, global retinal nerve fiber layer; HC, healthy control; INL, inner nuclear layer; mRNFL, macular RNFL; PwE, People with epilepsy; TCS, tonic–clonic seizure; TLE, temporal lobe epilepsy; TMV, total macular volumes.

### 3.3 Models for retinal neuroaxonal loss

**FIGURE 3:**
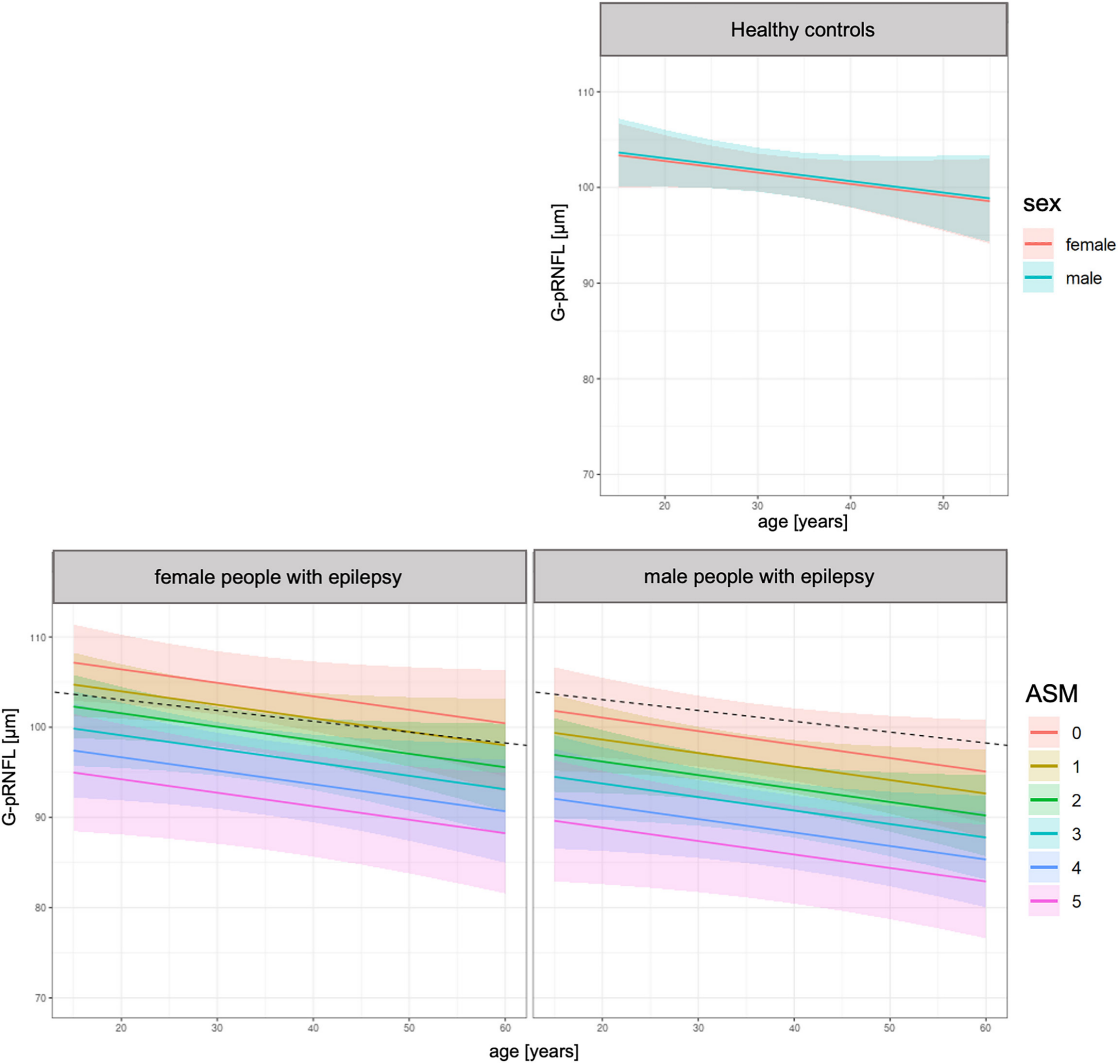
Age‐dependent G‐pRNFL thickness with regard to sex and the anti‐seizure medication (ASM) load. The graphs reflect the average and confidence intervals of the age‐dependent and sex‐specific global peripapillary retinal nerve fiber layer (G‐pRNFL) thickness in healthy controls (HC) as well as people with epilepsy (PwE). The graphs for the epilepsy cohort were calculated for a man and woman (body mass index: 25.8 kg/m^2^) with temporal lobe epilepsy with an annual frequency of six tonic–clonic seizures. For better group comparison, the average G‐pRNFL thickness of HC with a comparable age and sex distribution is also displayed as black dashed line. The G‐pRNFL was significantly thinner in PwE compared to HC, with more pronounced effects in men than in women. Further, the G‐pRNFL was thinner, the higher the number of anti‐seizure medications (ASM) taken at the time of study examination.

The results of the multivariate analyses are summarized in Table 3: according to our model, the G‐pRNFL was thinner the higher the number of current ASM, and the higher the maximum annual TCS frequency. Further, the G‐pRNFL thinning was more pronounced in case of male sex. Tests on collinearity were unsuspicious for any of the variables, i.e., the variance inflation factors were at maximum 1.6. Figure 3 summarizes the main findings on G‐pRNFL, i.e., the sex‐dependent G‐pRNFL thickness with regard to the number of current ASM.

**TABLE 3:** Selected models for the thickness and volume of the retinal layers in people with epilepsy.

Predictors	G‐pRNFL		mRNFL		GCIP		INL		TMV	
	Estimates	*P*	Estimates	*P*	Estimates	*P*	Estimates	*P*	Estimates	*P*
(Intercept)	110.25 (104.33 to 116.16)	**<0.001**	0.94 (0.88 to 01.01)	**<0.001**	2.16 (2.06 to 2.25)	**<0.001**	0.99 (0.96 to 1.03)	**<0.001**	**8.84 (8.72 to 8.97)**	**<0.001**
Sex: male	−5.35 (−8.64 to −2.06)	**0.002**			−0.04 (−0.09 to 0.01)	0.137				
Age	−0.15 (−0.31 to −0.008)	**0.062**	−0.001 (−0.003 to 0.0004)	0.123	−0.002 (−0.004 to −0.0007)	**0.160**	−0.001 (−0.002 to −0.0001)	**0.050**		
Max. annual frequency of TCS	−0.14 (−0.25 to −0.03)	**0.012**					0.0005 (−0.0002 to 0.0012)	0.142		
Number of current ASM	−2.44 (−4.03 to −0.84)	**0.003**	−0.03 (−0.05 to −0.005)	**0.015**	−0.04 (−0.07 to −0.01)	**0.006**			−0.09 (−0.15 to −0.03)	**0.003**
Neurostimulation therapy: yes			0.08 (0.008 to 0.15)	**0.031**	−0.089 (−0.19 to −0.013)	**0.085**	−0.04 (−0.07 to −0.003)	**0.033**		
Disease duration										
Observations	94		94		94		94		94	
*R* ^2^/*R* ^2^ adjusted	0.301/0.269		0.103/0.074		0.246/0.212		0.113/0.083		0.091/0.081	
	*F*‐statistic: 9.57 on 4 and 89 DF, *P* < 0.001		*F*‐statistic: 3.46 on 3 and 90 DF, *P* = 0.020		*F*‐statistic: 7.24 on 4 and 89 DF, *P* < 0.001		*F*‐statistic: 3.82 on 3 and 90 DF, *P* = 0.013		*F*‐statistic: 9.17 on 1 and 92 DF, *P* = 0.003	

*Note*: Bold indicates statistically significant value (*P* < 0.05).

Abbreviations: GCIP, ganglion cell inner plexiform layer; G‐pRNFL, global retinal nerve fiber layer; INL, inner nuclear layer; mRNFL, macular RNFL; PwE, People with epilepsy; TCS, tonic–clonic seizure; TMV, total macular volumes.

Similarly, all retinal layers except the INL were thinner the higher the number of current ASM. Neurostimulation therapy was thereby positively associated with the mRNFL volume and had a negative impact on the volumes of GCIP and INL. Disease duration had no significant impact on these retinal measures in the multiple linear model. The *R* ^2^ of the models, though, were small, suggesting that yet unconsidered parameters substantially contribute to the retinal neuroaxonal loss. The residuals of the model were close to a normal distribution.

In HC, a sex effect was observed for the GCIP (*β* = 0.01, *P* = 0.004) and INL (*β* = 0.03, *P* = 0.017) thickness, as well as the TMV (*β* = 0.18, *P* = 0.028) with higher measures in men compared to women. Of note, no sexual dimorphism was observed for the G‐pRNFL thickness. Age had no significant impact on the retinal measures.

## 4 DISCUSSION

This prospective OCT study systematically characterized the extent of the retinal changes in a broad range of well‐characterized adults with epilepsy, revealing a significant retinal neuroaxonal loss across the inner retinal layers (G‐pRNFL, mRNFL, GCIP, and INL), as well as a reduced total macula volume. This finding is in line with previous reports on RNFL thinning in adults with epilepsy,^11^, ^12^, ^13^, ^14^, ^16^ and complements the few available smaller‐scaled studies on GCIP,^11^, ^14^, ^18^ and TMV.^18^ Besides age as known driver of neuroaxonal loss in epilepsy,^12^, ^16^ we identified a significant association between the retinal neuroaxonal loss and the frequency of TCS, as well as the number of ASM. In addition, our findings suggest a sex‐specific vulnerability to the detriment of men.

### 4.1 Association between retinal neuroaxonal loss and clinical parameters

In our study, it was the presence and frequency of *TCS* that emerged as a relevant determinant of the retinal neuroaxonal loss. The retinal measures in PwE without TCS did not significantly differ from HCs even though they had a comparable disease duration, number of ASM and were significantly older than PwE with TCS. Their retinal measures were in‐between the HC group and PwE with TCS, suggesting more subtle retinal changes than in PwE with TCS. This analysis, though, was probably underpowered considering the small size of the subgroup without TCS (n = 19). Only one previous, smaller‐scaled (n = 43) OCT study also evaluated the impact of seizure frequency on the thickness of the inner retinal layers.^11^ However, they did not differentiate between seizure semiologies, what might have contributed to the negative study result. Our findings suggest that a significant retinal neuroaxonal loss primarily occurs in PwE with high disease activity and severity as reflected by the occurrence and frequency of TCS.

In addition, more active and severe epilepsies often require *polytherapy*, defined as a combination of at least two ASM. We thus hypothesize that the observed association of retinal neuroaxonal loss with the current number of ASM also reflects the impact of disease activity and severity. However, we cannot rule out that the ASM intake itself (besides seizure frequency and severity) contributes to the retinal neuroaxonal loss.

Likewise, *neurostimulation* should be understood as an indicator of severe and long‐standing epilepsy as it is typically used to treat patients with pharmacoresistant epilepsy who are no candidate or failed resection. However, the results on neurostimulation must be interpreted with caution, as the subgroup size was small, and the observed statistical effects limited and inconsistent between retinal layers. There is only one previous report on a negative univariate association of RNFL thickness and VNS implant.^12^ However, this was probably biased by a high collinearity between VNS and pharmacoresistant epilepsy.^12^

### 4.2 Sex‐specific vulnerability

Remarkably, the retinal neuroaxonal loss was significantly more pronounced in males compared to females with epilepsy – independent of age, disease duration, the number of ASM, and seizure frequency. In contrast, no significant sex‐specific effects were observed for pRNFL and mRNFL in our HC cohort. Of note, male HC revealed even significantly higher values for GCIP, INL, and TMV than female HC in our study. Thus, the retinal neuroaxonal loss associated with epilepsy might have been partially covered by the physiological sex‐specific differences. Our findings substantiate and extend a previous report on sex differences in RNFL thickness in PwE^12^ and are in line with a structural MRI study describing a larger vulnerability to seizure‐associated brain atrophy in men compared to women with TLE.^34^ Despite the increasing interest in sex‐specific differences in epilepsy, knowledge on sex‐specific effects and the underlying pathomechanisms is still scarce. Sexual dimorphism in epilepsy includes not only differences in cortical thickness and functional activity^34^, ^35^, ^36^ but also neurotransmitter signaling^37^, ^38^ and neurosteroid expression.^39^, ^40^ These factors might contribute to more severe peri‐ictal hypoxia and thus larger neuroaxonal damage in men, but their assessment was beyond the scope of our study.

### 4.3 Pathophysiological considerations

In PwE, the neuronal loss clearly exceeded the physiological expected, age‐dependent retinal involution, suggesting that epilepsy is associated with accelerated neuronal loss, which can be measured as retinal thinning. Its cause, though, remains to be elucidated. As argued above and in analogy to studies on brain atrophy in epilepsy,^30^, ^32^, ^41^ retinal neuroaxonal loss seems to reflect the disease activity and severity, i.e., the long‐term sequelae of repeated seizures and extensive therapies.

In the absence of further evidence, it remains speculative whether the observed retinal changes occur secondarily to seizure‐associated head trauma and cerebral neuroaxonal loss comparable to collision sport athletes,^42^ or whether cerebral and retinal changes are caused by the same (non‐traumatic) mechanisms and manifest in parallel. The latter seems more likely, considering that only a minority of TCS goes along with a relevant head trauma.^43^, ^44^ We hypothesize that the neuroaxonal loss is mainly driven by additional seizure‐accompanying mechanisms, above all the peri‐ictal hypoperfusion and hypoxia: an hypoperfusion can be found in the cerebral regions expressing seizure activity, not only during the late stage of seizures but also postictally. It is caused by vasoconstriction and accompanied by significant hypoxia,^45^ which might lead to an increased blood–brain barrier permeability, glial activation, central inflammation and neuronal loss. Of note, the peri‐ictal hypoperfusion/hypoxia is severer the longer the seizure duration^45^, ^46^ and positively associated with the extent of cerebral atrophy in epilepsy.^46^ Besides, Valsalva retinopathy might contribute to the observed retinal alterations.^47^ However, PwE report rarely visual loss or scotoma after an experienced TCS, i.e., the typical symptoms of Valsalva retinopathy, and it is thought to heal without traces. Thus, it seems unlikely that Valsalva retinopathy is a relevant determinant of the retinal alterations, except maybe in single cases or on a yet undescribed subclinical level.

Against this background, we hypothesize that the extent of the retinal neuroaxonal loss is explained as the cumulative sequelae of repeated peri‐ictal hypoperfusion/hypoxia events: in our study, (1) a significant retinal neuroaxonal loss was only observed in people with history of tonic–clonic seizures and (2) it was more pronounced in people prone to have longer or more frequent TCS, namely men^48^, ^49^ with increasing age.^50^

The pRNFL appears to be the first predilection site of retinal neuroaxonal loss with broadest associations with parameters of disease activity and severity. The GCIP and INL thinning might occur consecutively^9^, ^10^ in long‐standing severe epilepsies. However, longitudinal OCT studies are needed for further clarification.

### 4.4 Potential clinical relevance of the study findings

Considering that the retinal changes seem to reflect aspects of the disease activity and severity, the OCT measurement of the retinal layers could bear a great clinical potential for the diagnostic and treatment of PwE: In detail, the easy‐to‐handle, quick, cost‐effective, and non‐invasive OCT measurement could be included in the clinical routine to (1) provide the treating physician with an integrative measure of the individual disease burden. It does not only reflect the cerebral integrity and cognitive ability,^12^ but could (2) potentially identify persons at risk for cognitive decline, and (3) serve as a prognostic marker for surgery or neurostimulation outcome. Further, it might serve as a future tool to (4) assess the progression rate of the neuroaxonal loss, which could help to monitor the disease activity and treatment response. This could be of great importance in people who are not able to document their seizures to decide on treatment adjustments. The retinal thickness could thereby be an objective and more complex parameter than the so far used seizure frequency. Longitudinal OCT studies will be needed to determine the dynamic of the retinal changes and to prove the formulated hypotheses.

### 4.5 Limitations

The presented study encompasses a wide range of epilepsies, disease severity states, and drug regimens and is thus representative for the variety of patients managed in tertiary epilepsy centers. This setting also explains the relatively high percentage of people with multifocal epilepsies, because they are precisely characterized, which might have elevated the number of study participants with identified multifocal seizure onset zones. In addition, our center manages many people with difficult to treat epilepsies including those with neurostimulation, i.e., an epilepsy subgroup with an outstanding percentage of multifocal or undefined epilepsies. People with diagnosed genetic aberrations or syndromes (other than genetic generalized epilepsies) were not included in our study as this might have biased the pathophysiology and extent of neurodegenerative processes and should be evaluated separately. The downside of the variety of patient characteristics is, that it strongly limits subgroup analyses, although we performed an OCT examination of one of the biggest epilepsy‐control cohorts.

In consideration of the findings of prior OCT studies in epilepsy,^11^, ^12^, ^14^ we chose and studied an explorative set of parameters and their impact on neuroaxonal loss. We thereby confirmed and extended previous findings. However, the *R* ^2^ was always <0.3, indicating that our models only partially explain the observed retinal neuroaxonal loss and that other, yet unconsidered parameters substantially contribute to the retinal alterations. Further, seizure frequency was used as an estimate for disease activity. We are aware that seizure frequency is prone to underreporting due to postictal amnesia, cognitive impairment, and unrecognized nocturnal seizures.^2^, ^51^ However, there is still no tool available for reliable seizure detection that could have been used. Seizure detection devices might help to assess prominent motor seizures,^52^, ^53^, ^54^ but are still not capable of reliably detecting more subtle seizures, i.e., focal aware seizure (FAS), FIAS, and generalized non‐motor seizures. Moreover, an ophthalmological examination was not part of the study protocol. Instead, study participants were interviewed about visual aids, eye disease, and eye surgeries, and there was a comparable proportion of spectacle/contact lense wearers in the epilepsy (49%) and control (46%) group.

Further, HCs had significantly more years of education than study participants with epilepsy, most likely due to the recruitment from hospital staff and acquaintances. The different cognitive abilities, though, might have biased group comparisons, as the RNFL thickness is known to decrease with declining cognitive function.^55^ The intellectual ability, though, was not specifically assessed in our study, but people with cognitive impairment (as mentioned in medical records and/or IQ < 70 in previous neuropsychological testing) were excluded from the study. Due to organizational reasons, it was not possible to blind the OCT examiner (LD).

## 5 CONCLUSION

People with epilepsy are characterized by an extensive retinal neuroaxonal loss across the layers of the inner retina as well as the macula. Thereby, male patients on polytherapy experiencing frequent TCS seem to be at highest risk. OCT is a noninvasive, risk‐free technique that may provide a unique opportunity for studying pathophysiological mechanisms of the neuroaxonal loss in epilepsy. However, longitudinal studies are still needed to assess the significance of the retinal changes for therapy monitoring and patient management.

## AUTHOR CONTRIBUTIONS

LD collected the data, conducted statistical analyses, drafted the manuscript, and designed figures 1 and 2; HB and ML performed statistical analyses, created figure 3, and critically revised the manuscript; LS, GN, JC, and SN contributed to the patient recruitment and critically revised the manuscript; JH helped in the study design, advised the OCT acquisitions and analyses, and critically revised the manuscript; EK was responsible for the study design, data analysis, as well as manuscript drafting and revision.

## FUNDING INFORMATION

This research did not receive any specific grant from funding agencies in the public, commercial, or not‐for‐profit sectors. J.H. was (partially) funded by the German Federal Ministry of Education and Research [Grant Numbers 01ZZ1603[A‐D] and 01ZZ1804[A‐H] (DIFUTURE)]. The NeuroVisionLab (JH) was partially funded by grants for OCT research from Merck and Horizon.

## CONFLICT OF INTEREST STATEMENT

We confirm that we have read the Journal's position on issues involved in ethical publication and affirm that this report is consistent with those guidelines.

## FINANCIAL DISCLOSURES

L. Delazer, H. Bao, L. Stauner, G. Nübling, and J. Conrad report no disclosures relevant to the manuscript. M. Lauseker received honoraria from Celgene and financial support for research from Novartis, all outside of the submitted work. J. Havla reports grants from Friedrich‐Baur‐Stiftung, Merck, and Horizon; personal fees and nonfinancial support from Alexion, Horizon, Roche, Merck, Novartis, Biogen, B.M.S., and Janssen; and nonfinancial support from the Guthy‐Jackson Charitable Foundation and The Sumaira Foundation. S. Noachtar received speaker honoraria and financial compensation for travel expenses from Medtronic, UCB, Desitin, GlaxoSmithKline, Sanofi‐Aventis and Eisai, has participated in advisory boards and clinical trials for Desitin, Eisai, Medtronic, Pfizer, UCB, Glaxo‐Smith‐Kline, Pfizer, and Precisis and received financial support for research from Deutsche Forschungsgemeinschaft (DFG) (NO 419/2‐1), Bundesministerium for Bildung und Forschung (BMBF) (16Meo185) and Hertha‐Riehr‐Stiftung, all outside the submitted work. E. Kaufmann received speaker honoraria and financial compensation for travel expenses from Medtronic, UCB, Livanova, and Eisai and has participated in clinical trials for Medtronic, UCB and Precisis, all unrelated to the submitted work. She is partially funded by the Munich Clinical Scientist Program (MCSP).

## Data Availability

The data that support the findings of this study are available on reasonable request from the corresponding author. The data are not publicly available because it contains information that could compromise the privacy of research participants.
